# Warfarin induced mesenteric and intestinal hematoma requiring surgical resection to relieve small bowel obstruction: A case report

**DOI:** 10.1016/j.ijscr.2018.10.010

**Published:** 2018-10-12

**Authors:** Khuram Khan, Saqib Saeed, Sara Alothman, Farhana Iqbal, Alexius Ramcharan, Brian Donaldson

**Affiliations:** a Department of Surgery, Columbia University College of Physicians and Surgeons at Harlem Hospital Center, New York, 10037, USA; b Department of Medicine, Richmond University Medical Center, Staten Island, New York, USA

**Keywords:** Mesenteric, Intramural hematoma, Abdominal pain, Small bowel obstruction, Warfarin, Anticoagulant therapy

## Abstract

**Introduction:**

Mesenteric and Intramural Hematoma leading to small bowel obstruction is a rare, but a fatal complication of anticoagulant therapy.

**Presentation of case:**

We present this unique case of 61 year old male with non traumatic mesenteric and intramural hematoma secondary to warfarin leading to small bowel obstruction requiring surgical resection.

**Discussion:**

Although there have been few cases reported in literature about intramural small bowel hematoma, however, associated with small bowel obstruction secondary to warfarin therapy are less frequently seen. Diagnosis of intramural hematoma can be made with Computed Tomography (CT) of abdomen. The most effective treatment approach is conservative management after excluding any signs of small bowel obstructions, peritonitis and bowel necrosis.

**Conclusion:**

Therefore, early diagnosis is vitally essential to avoid morbidity in patients with these symptoms. Physician should have a higher index of suspicion to recognize and diagnose this complication is essential for early treatment intervention to prevent morbidity.

## Highlights

• Prolonged use of Anticoagulant therapy causing intramural small-bowel hematoma and ischemic bowel disease.
• Patients present with abdominal pain.
• CT scan is used to make the diagnosis.
• Physician should have a higher index of suspicion to recognize and diagnose this complication.
• Patients with a history of prolonged anti-coagulants should be monitored closely with prolonged INR values, especially in patients presenting with abdominal tenderness.
• Due to the rarity of this complication, more identification of such cases, observation and close surveillance is necessary.
• Early diagnosis and appropriate management with surgical resection to prevent morbidity.

## 1 Introduction

Anticoagulant therapy is widely used as prophylactic agent and treatment for assortment of thromboembolic and coagulation disorders. There are various potential complications related to anticoagulants, therefore, patients are required to monitor frequently. A rare but life threatening complication of anticoagulant therapy is spontaneous intra-abdominal hematoma. According to the literature, intramural small bowel hematoma is commonly seen secondary to blunt abdominal trauma.

The rarity of intra-abdominal hematoma and complications secondary to anticoagulants uses occur in about 1 case per 2500 patients on anticoagulant therapy per year [3]. All bleeding complications are seen during anticoagulant therapy, however, the incidence of intra-abdominal/intramural hematoma and or hemorrhage of small bowel is reported only in 2–4% of patients [6]. Warfarin induced intramural hematoma should be suspected as a complication if a patient presents abdominal pain, this makes early diagnostic and therapeutic interventions very crucial to prevent severe complications. This work has been reported in line with the SCARE criteria [1].

## 2 Case presentation

**Fig. 1:**
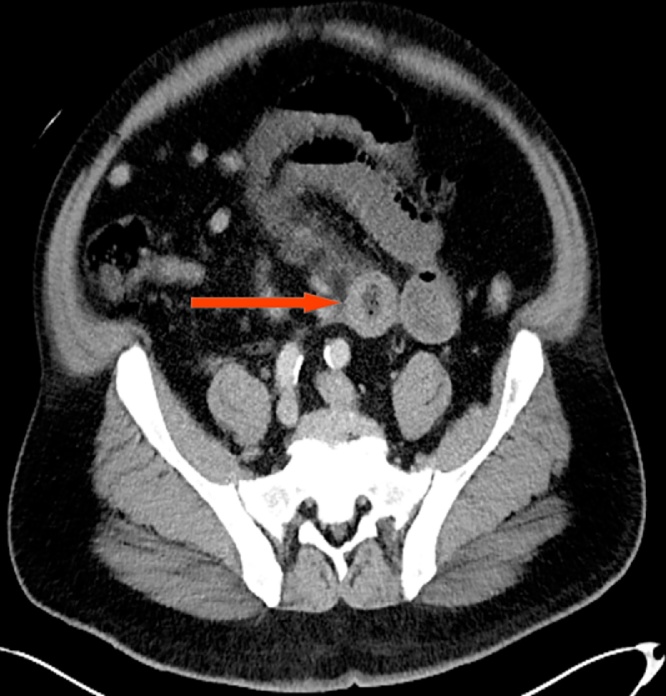
Computed Tomography of abdomen axial view showing dilated small bowels (target sign) (red arrow).

**Fig. 2:**
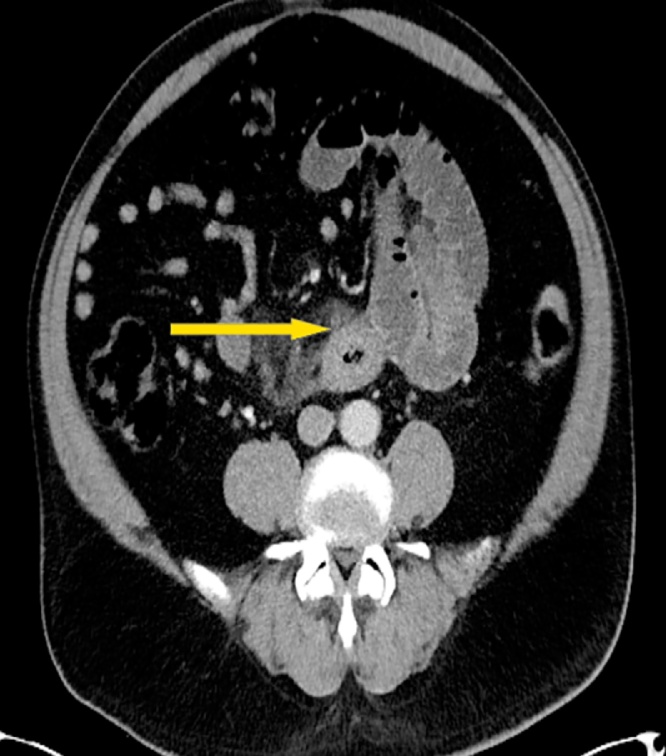
CT abdomen axial another view showing dilated small bowels along with mesenteric swirling; target sign (yellow arrow).

**Fig. 3:**
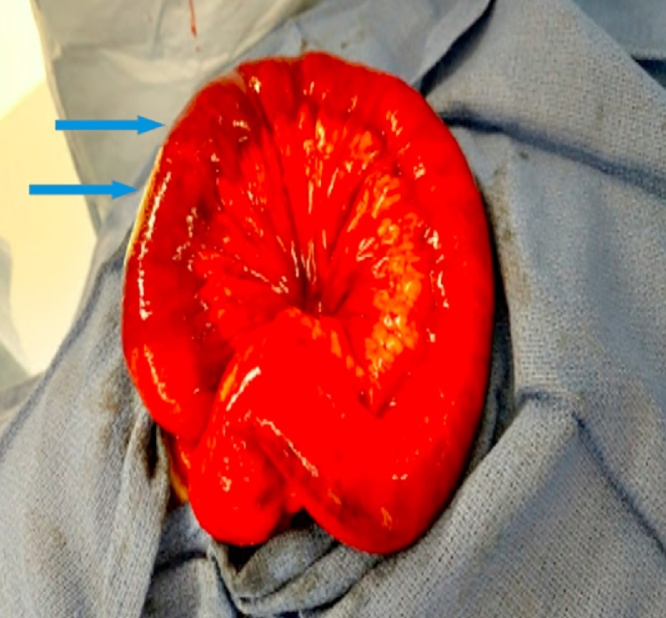
Gross image showing dilated small bowels along with intramural and mesenteric hematoma (blue arrows).

**Fig. 4:**
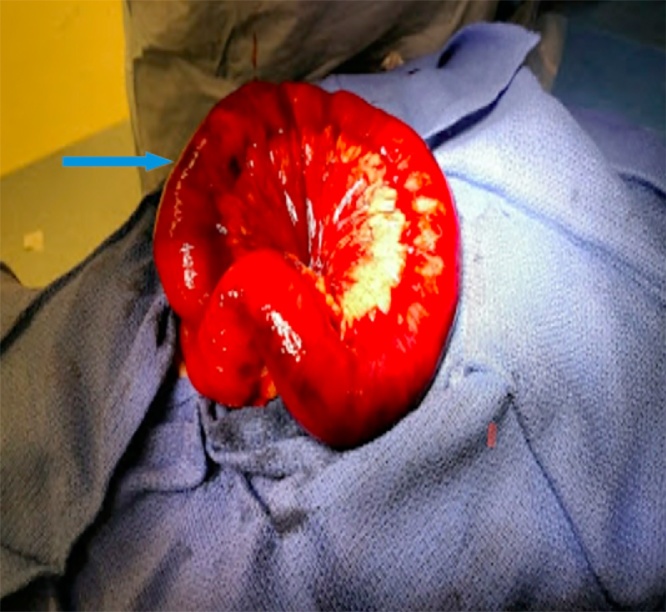
Resected gross image showing intramural and mesenteric hematoma (blue arrow).

A 61 year old male with history significant for right lower extremity deep venous thrombosis (DVT); on warfarin 7.5 mg, hypertension, diabetes mellitus, asthma, and chronic kidney disease presented to the emergency department with 3 day history of generalized abdominal pain associated with multiple episodes of nausea, vomiting and obstipation. Patient was awake, alert and oriented. Blood pressure was elevated, rest of vitals were benign. On physical examination, patient had distended abdomen with mild generalized tenderness, no signs of peritonitis. Remainder of his exam was un-remarkable. Labs were significant for INR; >6 and PTT; 91.9. CT abdomen was obtained that showed high grade small bowel obstruction (Fig. 1, Fig. 2). Patient was admitted under surgical team and initially managed by bowel rest with nil per os(NPO), nasogastric tube to suction showed 1.3 L of bilious fluid, and foley catheter was inserted. Patient was given 6 FFPs to resuscitate in the emergency room. Repeated INR obtained and was found to have 2.16. At this time, patient was taken to the operating room for diagnostic laparoscopy. Intra- operatively, patient was found to have dilated small bowel along with a segment of bowel with intramural and mesenteric hematoma involving 30 cm of the jejunum (Fig. 3, Fig. 4). Ischemic small bowel with intramural hematoma was resected and anastomosed primarily. Post-operative management in intensive care unit (ICU) was uneventful and patient was discharged home on post op day 5 without any further complications.

## 3 Discussion

Spontaneous intramural and mesenteric intestinal hematoma secondary to anticoagulant therapy are rare but fatal complication reported in medical literature [7]. According to a retrospective epidemiological study in a literature, the incidence of intramural small-bowel hematoma is at 1 case per 20,000 admissions to medical and surgical services or 1 case per 2500 patients per year with a history of anticoagulants use [4]. The etiology for the formation of intramural hematoma is rupture of the end artery which leads to muscular layer of intestine leading to formation of the hematoma [3].

In most literature it is reported that complication of small bowel hematoma and bleeding in patient with anticoagulant therapy is most commonly cited risk factor is severe hypertension [6]; our patient had a history of hypertension and on initial vitals presented with hypertensive urgency.

The spectrum of clinical symptoms may vary from mild abdominal pain followed by nausea/vomiting to severe intestinal obstruction, peritonitis, necrosis and acute abdomen. Rarely, patient may also present with hemorrhage, hematemesis, rectal bleeding and or melena [4]. In few reported literatures INR is found above the therapeutic range, in this case patient has INR > 6. It is thought that patients with a history of anticoagulant therapy who present with abdominal tenderness and abnormal elevated level of coagulation profile (elevated INR and PTT) should raise suspicion of small-bowel hematoma.

Currently, the initial diagnostic radiographic approach to evaluate intramural small-bowel hematoma is abdominal ultra sonogram with a confirmatory Abdominal CT scan [2]. Characteristic CT scan findings are symmetrical thickening and dilatation of the affected bowel and mesenteric engorgement with some luminal narrowing. In this case patient presented with significant dilated small bowels along with mesenteric swirling showing a target sign (Fig. 2). Most common sites of intramural small intestinal hematoma are jejunum and the ileum followed by duodenum as evident in our patient who was found to have dilated small bowel along with a segment of bowel with Intramural and mesenteric hematoma involving 30 cm of the jejunum (Fig. 3, Fig. 4).

Since the intramural small-bowel hematoma secondary to warfarin is rarely seen, there are no evidence based literature suggesting appropriate treatment. The most effective intervention recommended is conservative management after excluding any signs of small bowel obstructions, peritonitis and bowel necrosis. In the event of small bowel obstruction, immediate intervention and appropriate surgical resection of the obstructed bowel are essential. In this case, after initial resuscitation surgical intervention with diagnostic laparoscopy of the obstructed small bowel segment with intramural hematoma was resected and anastomosed primarily.

## 4 Conclusion

This case report illustrates a unique entity of prolonged use of anticoagulant therapy causing intramural small-bowel hematoma and ischemic bowel disease. Physician should have a higher index of suspicion to recognize and diagnose this complication is essential for early treatment intervention to prevent morbidity. Patients with a history of prolonged anti-coagulants should be monitored closely with prolonged INR values, especially in patients presenting with abdominal tenderness. Due to the rarity of this complication of warfarin anticoagulant therapy, evidence in literature is scarce about appropriate management. More identification of such cases, observation and close surveillance is necessary to find the pathogenesis, early diagnosis and appropriate management to prevent unnecessary surgical approach and morbidity with favorable outcomes.

## Conflict of interest

No conflicts.

## Sources of funding

No source of funding.

## Ethical approval

Ethical approval is not required by our institution. This was a case report and permission and consent has been taken from the patient.

## Consent

We have obtained a written informed consent from the patient for publication of this case series report and also accompanying images. A copy of the written consent is available for review by the Editor-in-Chief of this journal on request.

## Author contribution

Khuram Khan- Abstract, Figure collections, writing, format.

Saqib Saeed- writing, editing, others.

Sara Alothman- others, writing, format.

Farhana Iqbal- writing, editing, figure collections.

Brian Donaldson-review, final writing, editing.

Alexius Ramcharan- other, review, editing.

## Registration of research studies

It's case report. Nothing to register.

## Guarantor

Khuram Khan.

## Provenance and peer review

Not commissioned, externally peer reviewed.
